# Targeted drug delivery for cancer therapy: the other side of antibodies

**DOI:** 10.1186/1756-8722-5-70

**Published:** 2012-11-09

**Authors:** Michael A Firer, Gary Gellerman

**Affiliations:** 1 Department of Chemical Engineering and Biotechnology, Ariel University Center, Ariel, 40700, Israel; 2 Department of Biological Chemistry, Ariel University Center, Ariel, 40700, Israel

**Keywords:** Targeted drug delivery, Therapeutic antibodies, Antibody-drug conjugates, Peptide-drug conjugates

## Abstract

Therapeutic monoclonal antibody (TMA) based therapies for cancer have advanced significantly over the past two decades both in their molecular sophistication and clinical efficacy. Initial development efforts focused mainly on humanizing the antibody protein to overcome problems of immunogenicity and on expanding of the target antigen repertoire. In parallel to naked TMAs, antibody-drug conjugates (ADCs) have been developed for targeted delivery of potent anti-cancer drugs with the aim of bypassing the morbidity common to conventional chemotherapy. This paper first presents a review of TMAs and ADCs approved for clinical use by the FDA and those in development, focusing on hematological malignancies. Despite advances in these areas, both TMAs and ADCs still carry limitations and we highlight the more important ones including cancer cell specificity, conjugation chemistry, tumor penetration, product heterogeneity and manufacturing issues. In view of the recognized importance of targeted drug delivery strategies for cancer therapy, we discuss the advantages of alternative drug carriers and where these should be applied, focusing on peptide-drug conjugates (PDCs), particularly those discovered through combinatorial peptide libraries. By defining the advantages and disadvantages of naked TMAs, ADCs and PDCs it should be possible to develop a more rational approach to the application of targeted drug delivery strategies in different situations and ultimately, to a broader basket of more effective therapies for cancer patients.

## Introduction

Several potholes mark the winding road leading to the introduction of therapeutic monoclonal antibodies (TMAs) into routine clinical practice. Numerous excellent reviews have covered this period, dealing with the history of hybridoma technology, the development of monoclonal antibodies and their establishment as therapeutic agents [1-4]. Notwithstanding current challenges, there is justified continual development of and increased commercial interest in TMAs, testament to the diligence and capabilities of many scientists and engineers in laboratories around the globe. With the recent approval of Pertuzumab in June of this year, the FDA had now registered twelve TMAs for cancer therapy (http://lifesciencedigest.com/2011/03/05/fda-approved-mabs-for-cancer-therapy). Five of these are approved for hematological cancers. A number of hurdles remain to be overcome if TMAs are to become more effective and economic cancer therapies. These include selection of true cancer cell specific antigens, enhanced recruitment of bystander cell killing mechanisms, and development of more economic production technologies.

TMAs have mostly been used as naked antibodies but there is now high expectation of their employment in Targeted Drug Delivery (TDD), mainly because TDD systems can overcome many of the non-specific side effects associated with traditional cancer chemotherapy [5]. Indeed it is predicted that within the field of TMAs, the development of more effective Antibody-Drug Conjugates (ADCs) will be the focus of many biotech and pharmaceutical R&D programs over the near term. Nonetheless, given the importance of TDD and the challenges noted above, it is prudent to ask whether under some circumstances, TMAs may not be the most appropriate drug carrier. This article will discuss such situations and ask whether Peptide-Drug-Conjugates (PDCs) may be more appropriate alternatives to ADCs? First, we present an overview of the biological aspects of FDA approved TMAs, particularly those used for hematological cancers. (For summaries of the clinical efficacies of these drugs the reader is referred to recent reviews [6-8]). Similarly, we then discuss ADCs, highlighting their potential advantages. These overviews set the background to highlighting several limitations in the use of antibodies as drug carriers, which leads us to consider alternatives to antibodies, focusing on peptides, and to show how PDCs can overcome some of the limitations of ADCs. We believe these discussions are timely because identifying the advantages and disadvantages of both ADCs and PDCs in particular situations should lead to a more rational development and application of TDD strategies and ultimately to a broader basket of effective therapies for cancer patients.

## Therapeutic monoclonal antibodies - a view from above

Since Nadler et al. reported the proof-of-concept that a monoclonal antibody against lymphoma cells could be effective in human cancer therapy [9] and the FDA approval of the first TMA (Orthoclone OKT3®) in September 1992, there has been a slow but steady introduction of additional antibodies into the clinic. Currently, 39 TMAs have received regulatory approval and are marketed (IMGT database, http://www.imgt.org/mAb-DB/index), of which 12 are used in cancer therapy (Table 1) Despite this apparently small number, over 500 clinical trials are currently testing more than 160 candidate TMAs for cancer intervention ([10], ClinicalTrials.gov), with over 70 of these being Phase III trials. Even though a number of these trials are testing the same TMA in different clinical settings and given that only about 50% of Phase III trials are completed successfully, we can optimistically expect to see at least several newer TMAs receiving regulatory approval for cancer therapy over the next one or two years.

**Table 1:** FDA approved therapeutic monoclonal antibodies for cancer therapy

**Generic name**	**Proprietary name**	**Target**	**Technology**	**Isotype**	**Additional manipulations**	**Year FDA approved**	**Approved clinical indication**
Rituximab	Rituxin®/ Mabthera®	CD20	Mouse Hybridoma	IgG1-kappa	Chimeric	1997	NHL; later CD20+CLL, FL, RA
Transtuzumab	Herceptin®	HER-2	Mouse Hybridoma	IgG1-kappa	Humanized	1998	HER-2^+^ MBC
Alemtuzumab	Campath®/ Mabcampath®	CD52	Rat Hybridoma	IgG1-kappa	Humanized	2001	CL L, T-cell Lymphoma
Ibritomomab tiuxitan	Zevalin®	CD20	Mouse monoclonal	IgG1-kappa	Conjugated to Yittrium-90	2002	NHL
Tositumomab	Bexxar®	CD20	Mouse monoclonal	IgG2a-lambda	Conjugated to I-131	2003	NHL
Cetuximab	Erbitux®	EGRF, HER-1	Mouse monoclonal	IgG1- kappa	Chimeric	2004	EGRF^+^ MCC
Bevacizumab	Avastin®	VEGF	Mouse monoclonal	IgG1- kappa	Humanized	2004	MCC
Panitumumab	Vectibix™	EGRF, HER-1	Human monoclonal	IgG2-kappa	Human	2006	MCC
Ofatumumab	Arzerra™	CD20	Human monoclonal	IgG1-kappa	Human	2009	Refractory CLL
Ipilimumab	Yervoy™	CTLA-4	Human monoclonal	IgG1-kappa	Human	2011	MMel
Pertuzumab	Perjeta™	EGFR2, HER-2	Mouse monoclonal	IgG1-kappa	Humanized	2012	BC

BC, Breast cancer; MBC_Metastatic breast cancer; NHL, Non-Hodgkin's Lymphoma; CLL, Chronic Lymphocytic leukemia; FL, Follicular Leukemia; RA, Rhematoid arthritis; MCC, Metastatic colorectal cancer; MMel, metastatic melanoma.

Aside from the welcome increase in the number of TMAs in clinical development, it should be noted that currently approved TMAs, as well as most of those undergoing clinical assessment, were developed through the traditional process, beginning with the production of a murine monoclonal antibody which was then adapted for clinical use by chimerization or humanization. In recent years however, TMAs produced through newer technologies such as phage display libraries or transgenic mice have entered clinical trial and this trend is likely to be strengthened as these tools are further enhanced and validated. One example of this new generation of TMAs is Mapatumumab, discovered through the phage display technology of Cambridge Antibody Technology Ltd. Mapatumumab is a humanized IgG1 antibody targeting the TRAIL R1 antigen and is currently undergoing several Phase II assessments for advanced cervical cancer ([11], Clinicaltrails.gov).

Experience has shown that successful development of TMAs requires more that the technical capability to genetically engineer chimeric or humanized antibodies. These developments have proven critical in reducing the human-anti-mouse antibody immune response of the first TMAs, in increasing blood TMA half-life and in improving conscription of immune mechanisms. But it is now evident that a more detailed understanding of both the biology of the target cancer as well as the immune and non-immune effector tasks the antibody is expected to perform can significantly impact on the required molecular properties of the antibody. This was amply illustrated in the case of IgFc glycosylation, where it was shown that the fucose units in the polysaccharide attached to the CH2 heavy chain domain significantly affected antibody-dependant cellular cytotoxicity (ADCC). Engineered IgG antibodies lacking fucosylated oligosaccharides induce enhanced ADCC activity both in vitro and in vivo [12]. Additional preclinical knowledge about antigen distribution in target versus normal tissues, antibody pharmacokinetics, efficacy in different models of the target disease and antibody uptake into (solid) tumor tissue is essential for the rational selection of lead antibody candidates, choice of appropriate patient study groups and effective design of expensive clinical trials. Considerations of downstream manufacturing of the final product must also not be forgotten, as these influence not only process economics but also the molecular structure of the product [13,14].

## TMAs for hematological cancer

The current picture of TMAs for hematological malignancies parallels that seen with cancer immunotherapy in general in that only a limited number of antibodies have been approved, although a number of candidates are undergoing clinical assessment. Of the 12 TMAs approved for cancer therapy, 5 are employed for leukemia or lymphoma therapy (Table 1).

### Rituxin

Rituxin, or Rituximab, was approved for the treatment of patients with Non-Hodgkin’s B-cell Lymphoma (NHL) in 1997 [15] and was actually the first TMA to be approved for cancer therapy. Rituxin is a chimeric antibody targeting CD20, a 33-35kDa cell-surface glycosylated phosphoprotein. Expression of CD20 is found on late-stage pro-B-cells (CD45R^+^/CD117^+^) and this expression increases with B-cell maturity, although it is absent from plasma cells [16]. CD20 was found overexpressed in several types of leukemias [17]. While no natural ligand has yet been described, its function may be related to effective B-cell responses to T-cell independent antigens, although other evidence suggests that CD20 acts as a calcium ion channel [16]. Early studies [18] demonstrated that Rituxin could induce B-cell depletion by several immune effector mechanisms including ADCC and complement-dependant cytotoxicity (CDC). Its ability to induce apoptosis has not been clearly demonstrated. Surprisingly, despite the pleiotrophic B-cell expression of CD20, B-cell lymphopenia does not seem to be related to increased rates of infection in long-term treated patients. This may be due to the ability of hematopoietic stem cells to regenerate the B-cell population relatively quickly. Rituxin does induce infusion reactions, possible resulting from release of inflammatory cytokines following administration [19]. Rituxin is currently employed in the management of several forms of NHL including Chronic Lymphocytic Leukemia (CLL), Follicular Lymphoma (FL) and Diffuse Large B-cell Lymphoma, most often in combination with chemotherapies [6]. Its use has had a profound effect on the management of patients, becoming the standard of care in NHL and FL, as well as in CD20^+^ CLL.

### Anti-CD20-radioisotope conjugates

Ibritumumab is a murine anti-CD20 monoclonal antibody conjugated to the yttrium isotope (90Y-Ibritumumab tiuxetan). This intense β-radiation releasing immunoconjugate was approved in 2002 for use in patients with NHL but has also shown efficacy in Rituxin-refractory lymphoma [20]. Another immunoradioisotope, tositumomab-I^131^, was approved in 2003 for treatment of patients with CD20+ FL. Both drugs are efficacious but induce hemato-toxicity and have been the subject of several comparison clinical trials [21-23].

### Newer anti-CD20 TMAs

Despite its proven clinical benefit, clinical response rates to Rituxin are still modest, at about 50% for NHL [24]. The even lower figures reported for CLL [25] likely reflect the variable expression of CD20 on the surface of these tumor cells as compared to some other forms of B-cell leukemia. Possibly because more promising B-cell leukemic markers have not yet been discovered, and also as a result of ongoing research into the biology of anti-CD20 antibodies, much effort has been invested recently in overcoming some of the limitations of these first generation antibodies. These include binding-induced modulation of CD20 expression [26], diverted binding to blood forms of free antigen [27], relatively low binding affinity [28,29], development of resistance [28], genetic variability in the FcγRIIIa receptor gene among patients that affects ADCC activity [30] and uncertainty as to the mechanism of action. Studies on anti-CD20 antibodies has allowed their classification into Type 1 and Type 11 categories, based on their ability to induce changes in the membrane distribution and configuration of the antigen. These changes are thought to be related to the functional characteristics of the antibodies, such as their ability to form CD20/anti-CD20 lipid rafts, a function that may enhance their therapeutic efficacy [31]. In addition, while Type I antibodies effectively conscript CDC and ADCC, they are weak in inducing direct tumor cell death. They also modulate CD20 antigen expression, especially on CLL and mantle cell lymphoma cells [26]. On the other hand, Type II antibodies strongly induce direct tumor cell death , while inducing lower CDC but higher ADCC activity than Type I antibodies (reviewed in [16]). It should also be noted that while the tumor cell cytotoxicities of ADCC and CDC can be demonstrated, the technical validation of both the in vitro and in vivo methods is not-trivial [32-34]. In addition, while NK cells, important effectors of ADCC, seem to infiltrate most types of solid tumors [35], it is unclear whether at least under some situations they also have a tumor promoting effect, [36]. Further understanding the molecular differences between Type 1 and Type 11 antibodies that might account for these differences in effector mechanisms should allow the engineering of more potent antibodies as recently discussed [37]. Several next-generation anti-CD20 TMAs are now discussed below.

#### Ofatumumab

This IgG1 molecule is considered as a second-generation, fully humanized, anti-CD20 antibody and approved by the FDA in 2009 for treatment of CLL patients resistant to both fludarabine and alemtuzumab. It is currently being further tested in 9 Phase III intervention trials for leukemia (Clinicaltrials.gov). Like Rituxin, Ofatumumab is a Type 1 antibody but it recognizes a different CD20 epitope which seems to be related to its ability to induce high CDC responses, which may help explain is activity against Rituxin-resistance cells [38,39]. Treatment with Ofatumumab results in a list of side effects including neutropenia and increased risk of infections, but clinical data support its use both as monotherapy and in combination therapies [40].

#### Veltuzumab

Veltuzumab is another Type 1, humanized, anti-CD20, IgG1 mAb which binds to a similar region of the antigen as does Rituxin. The only difference in CDR structure is a single Asp101 to Asn101 switch in the CDRH3 region which may account for its higher binding affinity than Rituxin, but does not explain its improved CDC activity [41]. Veltuzumab is currently under development for the treatment of NHL, CLL and autoimmune diseases (Clinicaltrials.gov) [42].

#### Ocrelizumab

Ocrelizumab, another Type 1 humanized, anti-CD20, IgG1 antibody, also recognizes a similar epitope to that of Rituxin. It is a new generation antibody in that the Fc region has been engineered to increase binding affinity for the FcγRIIIa receptor, leading to enhanced ADCC but reduced CDC activities [43]. While some studies have tested its efficacy in hematological cancers [44], it has mostly been investigated for treatment of multiple sclerosis and rheumatoid arthritis [45].

#### Next-generation anti-CD20 antibodies

A series of newer, experimental anti-CD20 antibodies (e.g. PRO-131921 and AME133v) are also in development, of which the most clinically advanced is GA-101 (Obinutuzumab). This is a Type II, humanized antibody that has been glycol-engineered in CHO cells and differs significantly from previous anti-CD20 antibodies in its glycosylation of Asp297 in the CH2 Fc region endowing the antibody with enhanced ADCC but reduced CDC activity [46,47]. While GA-101 recognizes an epitope similar to that of Rituxin and Ocrelizumab, point alterations in its variable region sequences result in increased binding affinity. There are currently 9 Phase 1, II and III trials investigating this antibody in B-cell leukemias (Clinicaltrials.gov).

### Targets other than CD-20

#### Alemtuzumab

This fully-humanized IgG1 antibody targets CD52, a glycosylphosphatifylinosital-anchored cell surface glycoprotein expressed on both normal and malignant T and B lymphocytes, as well as on several myeloid-derived cells such as NK, macrophages and eosinophils [48]. It received FDA approval in 2001 for treatment of CLL with relapsed or refractory disease and for previously untreated CLL patients in 2007. Therapy with this antibody can induce T and B lymphopenia and immunosuppression so treatment must be accompany antibiotic and antiviral prophylaxis. Alemtuzumab seems to invoke by both ADCC and CDC but also directly induces apoptosis [49].

#### Milatuzumab

This humanized antibody targets CD74, an integral protein overexpressed in B-cell leukemias. The antibody is currently undergoing assessment in 5 Phase 1/II trials. In a pre-clinical study of mantle cell lymphoma the combination of Milatuzumab and Rituxin gave significantly enhanced therapeutic activity [50].

The above overview shows that the development of TMAs for hematological malignancies is still a very active area of basic and translational research. Predictably, these types of cancers are more amenable to immunotherapy where the antibodies can bypass problems faced in the treatment of solid tumors, such as tissue penetration (see below). Clinical response rates with these drugs vary widely between 35-75%, depending on the clinical setting and whether they are used as monotherapy or in combination with chemotherapy [6]. None of these antibodies target cancer cell specific antigens and a breakthrough development in this area would certainly enhance their clinical efficacy and reduce side effects.

## Antibody drug conjugates (ADCs)

Parallel to the development of TMAs has been the emergence of ADCs. ADCs bring together the targeting advantages of antibodies with the cytotoxic potential of chemotherapy, heralding the promise of targeted accumulation of drug in the tumor tissue. Although the idea to combine these qualities came early on in TMA development, creating clinically successful ADCs has proven difficult. Early attempts focused on Rituxin-Doxorubicin conjugates but these showed little clinical efficacy. Over time, it became clear that at least two factors are essential in the development of successful ADCs. The first is drug potency. From studies with early ADCs it became clear that very low amounts of antibody actually become deposited inside solid tumors [51], probably due to slow mass transfer and also that the deposition is uneven across the tumor tissue [52]. To overcome this limitation, drug candidates with potency several orders of magnitude higher than those used in conventional chemotherapy were selected. Examples of drugs currently used in ADCs include microtubule inhibitors such as the uristatins and maytansinoids and DNA-damaging agents like calicheamycin or duocarmycin analogs (reviewed in [53,54]). The second factor is the design of appropriate linker molecules for coupling drugs to the antibody. These not only must maintain antibody binding capacity following conjugation, but also should undergo selective enzymatic or chemical degradation inside the cell or at the cell surface, rather than systemically. In view of the extremely enhanced drug potency, this is essential if collateral damage is to be kept to a minimum. These points are discussed in more detail below.

To date, only 2 ADCs have been FDA approved for cancer therapy; only one of these is currently in clinical use. As the problem with tumor penetration by full length antibodies should be less important for hematological cancers, it is probably not surprising that the first ADCs targeted these malignancies (Table 2).

**Table 2:** **FDA approved Antibody**-**Drug Conjugates and selected others undergoing clinical development for cancer therapy**

**Generic name**	**Trade/code name**	**Target**	**Antibody source**	**Antibody isotype**	**Other manipulations**	**Conjugated to**:	**Clinical status**	**Indication**
Gemtuzumab ozogamicin	Mylotag®	CD33	Mouse monoclonal	IgG4- kappa	Humanized	Calicheamicin	Approved 2000 WITHDRAWN 2010	CD33+AML
Brentuximab vedotin	Adcentris™	CD30 (TNFR)	Mouse monoclonal	IgG1- kappa	Chimeric	Monomethyl auristatin E (MMAE)	Approved 2011	HL
Trastuzumab emtansine	MCC-DM1/ T-DM1	HER-2	Mouse monoclonal	IgG1- kappa	Humanized	Maytansinoid DM1	Phase III	HER-2^+^ MBC
Inotuzumab ozogamicin	CMC-544	CD22	Mouse monoclonal	IgG4- kappa	Humanized	Calicheamicin	Phase III Phase II	NHL DLBCL
Lorvotuzumab mertansine	IMGN901	CD56	Mouse monoclonal	IgG1- kappa	Humanized	Maytansinoid DM1	Orphan Drug 2010; Phase II	SMLC, OC, MM
------	SAR3419	CD19	Mouse monoclonal	IgG1	Humanized	Maytasinoid DM4	Phase I	NHL

Legend: AML – Acute myologenous leukemia; HL – Hodgkin’s lymphoma; NHL – Non Hodglin’s Lymphoma, MM – Multiple Myeloma; DLBLC – Diffuse large B cell lymphoma; OC – Ovarian cancer; MBC – Metastatic breast cancer.

### Gemtuzumab ozogamicin (Mylotag)

Gemtuzumab is a recombinant, humanized IgG4 monoclonal antibody (mAb) targeting CD33, an antigen expressed on most leukemic blast cells but also on normal hematopoietic cells, although the intensity of expression diminishes with normal stem cell maturation. The antibody is linked to calicheamycin and was the first ADC approved by the FDA (in 2000), for use in patients with relapsed acute myelogenous leukemia [55]. Almost from the outset however, the use of Mylotag was associated with significant side effects [56,57] and it was eventually withdrawn by its developers Pfizer in June 2010.

### Brentuximab vedotin (Adcetris)

This ADC consists of a chimeric antibody directed to CD30, a member of the tumor necrosis factor receptor family. CD30 is rarely expressed on T or B-cell lymphomas, but is a tumor marker for classic Hodgkin's Lymphoma, anaplastic large cell lymphoma and embryonal carcinomas. The antibody is conjugated to the antimitotic compound monomethyl auristatin E. Adcetris was granted accelerated marketing approval in August 2011 and is the first new Hodgkin’s Lymphoma drug in 30 years [58,59].

A number of other ADCs are currently undergoing clinical assessment. Indeed a recent Biopulse survey of pharma and biotech companies clearly indicates that many drug manufacturers are actively involved in ADC development and expect that many more of their products will enter the clinic in the coming years (http://www.bptc.com/sites/default/files/biopulse_reports/adc_survey_results_2011-11-23.pdf). Selected ADC candidates are discussed here.

### Trastuzumab emtansine (T-DM1)

This conjugate is based on the well studied antibody Trastuzumab (Herceptin) that targets the HER-2 cell surface protein and which was approved in 1998 for use in patients with Her-2+ metastatic breast cancer. The extensive clinical experience with this antibody has aided in both selection of an appropriate drug (maytansine derivative DM1) and the conjugation chemistry. Following the positive results of several preclinical [60] and recent clinical trials [61], even in woman whose disease had progressed during naked Trastuzumab therapy [62], the developers (ImmunoGen/Genentech/Roche) plan to apply for FDA approval before the end of 2012.

### Inotuzumab ozogamicin (CMC-544)

The conjugate contains an IgG4 monoclonal antibody targeting the CD22 antigen found on mature B cells. It is also being developed by Pfizer that is using the same hydrazone linker–calicheamicin drug combination as employed for Mylotag. It was shown to be more effective in vitro than Mylotag in killing primary pediatric acute lymphoblastic leukemia cells [63]. Despite the high potency of the drug moiety, initial clinical studies suggest relatively low tolerable doses of the CMC-544 ADC [64]. CMC-544 is currently the subject of 15 clinical trials, including two Phase III combination trials studies with Rituxin.

### Lorvotuzumab mertansine (IMGN901)

The Lorvotuzumab humanized monoclonal antibody targets CD56, an isoform of neural cell adhesion molecule expressed on NK cells, some T-cells and on the majority of Multiple Myeloma and Small Cell Lung Carcinoma cells. Different chemistry has been used to prepare this ADC in that the antibody is coupled to mytansioid (DM1) via a linker cleavable by disulfide reduction. In 2010 it was granted orphan drug status both in the USA and Europe for Mantle Cell Carcinoma, and is currently also under clinical investigation for Multiple Myeloma, Small Cell Lung Cancer and Ovarian Cancer.

### SAR3419

CD19 is expressed on follicular dendritic cells. It is a validated marker of developing B cells which is lost on their maturation to plasma cells. SAR3419 consists of the humanized huB4 anti-CD19 antibody conjugated to a different derivative of mytansinoid (DM4), but as in IMGN901, the disulphide linker is cleaved by reduction. In vitro studies show that this ADC is internalized by lymphoid cell lines and Phase I studies indicate it produces low hematological toxicity [65]. It is being clinically assessed for use in several forms of B-cell NHL.

There are more than a dozen other ADCs at various stages of preclinical and early clinical development. Unfortunately, many of these still induce side effects often seen in conventional chemotherapy such as several manifestations of myelosupression and nephritis and further research is needed in this respect before ADC can fulfill their promise of inducing less morbidity than carrier-free chemotherapy. Further clinical details on the activity of these agents can be found in recent reviews [54,66-68].

## Limitations of Abs as drug carriers

Despite the encouraging preclinical and clinical data that is emerging on the use of ADCs for cancer therapy, a number of challenges remain for the successful clinical translation of these drugs, some of which might represent inherent limitations in the use of antibodies as drug carriers. The most important of these are listed in Table 3 and discussed below.

**Table 3:** Comparison between full length antibodies and peptides as drug carriers in targeted drug delivery

**Item**	**Antibodies (full length)**	**Peptides**
**Discovery of novel cell surface targets**	Most approved TMAs do not target TSAs; for traditional mAbs target must be antigenic; screening selects mAbs to dominant epitopes; mAb specs depend on strain mouse/rat used	Target does not need to be antigenic; no prior knowledge of target molecule needed
**Generation technology**	Traditionally via murine hybridoma, then humanization; humanized mouse; via phage scFv phage display then grafting to Ig backbone	Combinatorial DNA, RNA, peptide library phage or cell based display technologies (random or scFv based); Combinatorial chemistry
**Molecular structure**	Standard Ab unit; different Ig isotypes; bispecific Ab; multi-bodies	Linear; cyclic; scFv; non natural amino acids; novel small molecules
**Intracellular transport**	Not a selection criteria of currently approved TMAs; technically difficult to select during screening	Screening technologies allow for easy selection of candidates that induce rapid endocytosis
**Pharmacodynamics and Pharmacokinetics**	Non-linear, depends of many variables, difficult to predict	Smaller molecular mass; larger formulation knowledge base for designed PD and PK
**Conjugation of carrier to drug** (**for ADC or PDC**)	Only ~50% mAb bound to drug; difficult to predict mAb/drug stoichiometry and drug position; conjugation chemistry limited to aqueous solutions.	Enhanced flexibility in conjugation chemistry for coupling to linker and drug, allowing wider selection of drugs including non-water soluble compounds, synthesis in organic solvents and aqueous solutions ; scaffolds available for conjugation to different drugs; formation of metal complexes; defined and predictable products;
**Antigenicity of final product**	Depends of extent of humanization.	Negligible
**Bystander immune effector function**	ADCC; CDC; CTL?;	None
**Tumor penetration**	Limited in solid tumors	Enhanced
**Manufacture**/**Quality Control**	Structure of ADC heterogeneous; high upstream development, cell culture, bioreactor design) and downstream ( purification) costs	Significantly lower production costs (up to ~35 amino acids); increased product reproducibility

TSA – tumor specific antigen; mAb – monoclonal (hybridoma) antibody; scFv – single chain Fv region of antibody combining site; ADCC – Antibody dependant cellular cytotoxicity; CDC – Complement dependant cytotoxicity; CTL – Cytotoxic T-lymphocyte; Ig - Immunoglobulin.

### Discovering novel cancer cell specific antigens

Studies using classical biochemistry, gene and protein array technologies and now systems biology have demonstrated differences in the metabolism of tumor cells compared to normal, as well as normal proliferating cells [69-71], one consequence of which is a modified “membrane-ome”, manifested either as altered expression of differentiation and constitutive mature cell proteins or re-expression of neonatal ones. This phenomenon led to the discovery of an array of tumor associated cell surface antigens (TAAs) [72-74] Confidence in the ability to utilize antibodies to identify TAAs was based in part on the success of immunologists over the years at producing antibodies to a variety of different molecules, even when these were considered weak antigens [75]; indeed an impressive battery of antibodies to cell surface markers is now available. Nonetheless, one take-home message from this massive effort is that the vast majority of TMAs only target TAAs. This was also evident from studies using molecular cloning techniques such as SEREX (serological analysis of autologous tumor antigens by recombinant cDNA expression) in which a patient's own serum is analysed for immunoreactivity against proteins expressed by their own tumor cells [72,76]. Only rarely have true tumor specific antigens (TSAs) so far been demonstrated, particular across different patients, the best examples being clonotypic antibodies expressed on antigen-educated B-cell leukemic cells such as those found in B-cell lymphoma [77] and Multiple Myeloma although the prevalence of these among different patient cohorts such is still inconclusive [78]. Aside from these, the most tumor cell “restricted” antigen identified so far, with regards to approved TMAs, is human epidermal growth factor receptor 2 (HER-2 or Erb-B2), which is targeted by Trastuzumab. While TAA-directed TMAs clearly have clinical benefit, their lack of specificity for true tumor cell targets is intrinsically associated with side effects that limit their efficacy.

It is noteworthy that currently approved TMAs and many of those in advanced stages of clinical development were generated several years ago, using technologies that may inherently limit the scope of molecular or structural targets identified. For example discovery platforms based on traditional hybridoma technologies coupled with high-throughput or FACS screening methods tend to skew towards selection of antibodies targeting dominant epitopes [79]. In addition, epitope dominance also depends of the genetic background of the rodent strain used for immunization, although this variable is not often studied due to the incurring development time and costs. Other limiting factors include the accessibility of potentially useful epitopes within a native multi-molecular complex to bulky antibody proteins and the lower antibody binding affinities towards weaker antigens. It remains to be seen whether more recent technical improvements aimed at overcoming these variables [80] will yield clinically successful TMAs to novel tumor cell markers.

### The problem of internalization

While there are some exceptions [81], the majority of anti-cancer drugs act intracellularly. With regards to ADCs this means that the antibody must bind a cell surface component in such a way that stimulates the formers' internalization, usually by receptor mediated endocytosis and then delivery of the ADC to lysosomes. In many cases, internalization was not a criteria included in the original selection of TMAs currently in clinical use, although subsequent studies showed that some of them, such as Transtuzumab, do induce uptake [82]. The requirement for ADC internalization makes the selection of TMAs even more complex. For example CD19, a validated B-cell marker, forms dimers with CD21 [83]. Whereas some anti-CD19 antibodies are rapidly internalized and are being used to develop ADCs ([65] and see above), the uptake of others is inhibited by CD21 expression [84]. Similarly there are conflicting reports regarding anti-CD20 antibodies [82,85]. In addition, studies with anti-HER-2 antibodies suggest that internalization is also related to affinity. For example Rudnick and colleagues recently reported that higher affinity antibodies were internalized and degraded faster than moderate affinity antibodies, thus limiting their tumor penetration [86]. These studies underline the importance of early stage selection of antibody clones with the appropriate functionality.

### Pharmacology

A number of studies have investigated the complex pharmacokinetics and pharmacodynamics of TMAs, which tend to be non-linear as compared to small molecule drugs [52,87-89]. The optimal values for these parameters can vary widely between antibodies as they depend on a wide variety of variables including heavy chain isotype, type and degree of glycosylation, route of administration, rate of absorption, antibody affinity, the location, cell surface density and turnover of antigen, charge, immunogenicity, ability to bind the FcRn receptor, effector function and degree of internalization.

### Attachment of linker-drug to antibody

**Figure 1:**
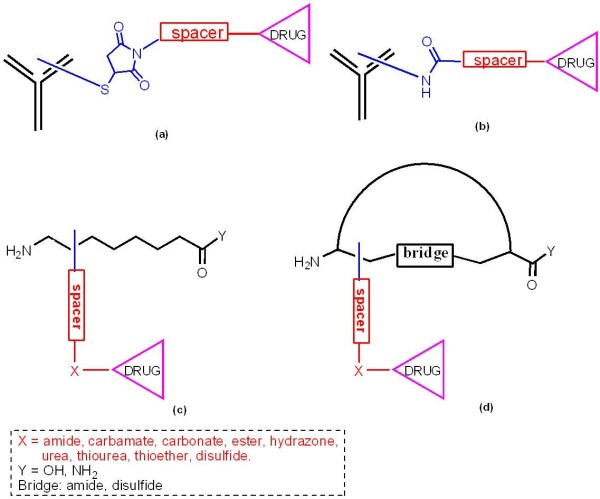
**Schematic representation of antibody**- **and peptide**-**drug conjugates.** ADC: (**a**) maleimido linker; (**b**) liner amino acid linker; For PDC: (**c**) liner peptide with spacer and bio-degradable group (X); (**c**) cyclic peptide with amide and disulfide bridge bearing spacer and bio-degradable group (X).

One area that has received much attention in ADC development is the chemistry of drug attachment. Factors important here include selection of a linker attachment site that retains antibody activity, linker length and composition and the design of drug analogs for attachment to the linker [51,53,54,82,90]. Two methods are commonly used for conjugating drugs to antibodies: alkylation of reduced interchain cysteine disulfides through an enzymatically non-cleavable maleimido or simple and cleavable disulfide linker (Figure 1a) and acylation of lysines by cleavable linear amino acids (Figure 1b). Spacers are usually essential extensions of the drug linkage and are responsible for avoiding the shielding of the active site of the antibody as well as improving solubility properties of ADCs (for example in by the use of polyethylene glycol). Cathepsin-cleavable linkers are also utilized (for example Val-Cit, or Phe-Lys) bound to self-emulative moiety PABA (*p*-aminobenzyl alcohol), enabling selective drug release in cancer cells [54], Notably, the linkage technologies used in ADCs are also applicable in PDCs enriching their conjugation repertoire as will be discussed later due course There are 8 interchain cysteines and up to 100 lysines available for conjugation on IgG1 antibodies and conjugation to these sites results in heterogeneous mixtures. Cysteine conjugates provide a greater degree of uniformity than lysine-based conjugates [91,92] while recombinant methods in which cysteines are introduced into the antibody backbone at specific sites result in still more uniform conjugation [93,94]. In some instances, it has been observed that the location of the conjugated drug is not as important as the stoichiometry of drug attachment, although this is difficult to ensure [91,93]. ADCs with two to four drugs per antibody are generally superior to more heavily loaded conjugates that tend to be cleared very rapidly from the circulation [95]. Nonetheless, it has been proved to be difficult to use chemical methods to prepare ADCs with a predefined (2, 3 or 4) number of drug molecules per antibody. Because the drugs are often conjugated to the side chains of reactive lysine or cysteine residues of the antibody, a distribution of products results in variable numbers of drug moieties attached to various sites. Thus, the formation of reproducible ADCs is difficult. Protein engineering has been used to circumvent these issues, such as inserting additional cysteine residues [94], or replacing solvent accessible cysteines with serines [93], although the drug loading stoichiometry still varies markedly so it is still difficult to control the number and regioselectivity of conjugated drug molecules.

### Drug bound versus free antibody

There is usually at least a 3 log_10_ difference in molecular weight between antibody and the linker-drug moiety. Therefore the conjugation of only 2–4 drug molecules produces a situation in which it is not a trivial task to separate the conjugated ADC from the unconjugated antibody. For example is was reported that in the formulation of the now discontinued Gemtuzumab Ozogamicin ADC (Mylotarg), only 50% of the anti-CD33 antibody was bound to 4–6 drug molecules. The remaining antibody was unconjugated [55]. This study underscores two problems. First, the presence of unconjugated antibody would presumably undermine the efficacy of the ADC. Second, the molar ratio of antibody:drug is an average value, indicating that the drug conjugation process produces a heterogeneous mixture of products with variable efficacy (discussed further below). This mixture would also not be exactly reproducible between batches.

### Tumor penetration

The limited penetration of full length antibodies into solid tumors is a recognized factor restricting their efficacy. Their large molecular size effects the rate of mass transfer due to higher interstitial pressure and hypoxia caused by leaky vasculature in the interior of the tumor mass, factors that can also effect levels of antigen expression [96-98]. It has however been possible to select antibody clones according to their degree of tumor penetration [86,99]. Interestingly, in those studies truncated (single chain variable fragment, scFv) and full length antibody penetration into the tumor mass was inversely related to the antibody affinity and internalization rate of the target antigen. These results are counterintuitive to the traditional strategy of developing high affinity antibodies and again highlight the need for a deep understanding of the biology of immunotherapy when selective TMA candidates. These issues seem less pertinent for micrometastases and even more so for hematological tumors such as CLL where the malignant cells are blood borne.

### Manufacturing issues

The current limited levels of TMA efficacy require the use of significant quantities of product (150-350mg/m^2^ single dose) over multiple treatments. This situation has stimulated an impressive expansion and improvement in the whole gamut of upstream and downstream processes involved in antibody manufacture (reviewed in [14]), including animal cell recombinant expression systems, cell culture media, cell growth conditions and product yield/cell. Several important challenges remain in the area of product quality control such as the production of aggregates [100] and variation in glycosylation [101]. Moreover, further improvements are urgently needed in bioreactor design and protein purification processes if overall manufacturing costs is to be lowered to enable TMAs and ADCs to be affordable for patients who need them [14,102].

TMAs have already proven their clinical effectiveness and it is predicted that the coming two years will see 1–2 ADCs receiving FDA approval, at least one of which will be for hematological cancers. Nonetheless, the points raised above indicate that both TMAs and ADCs have some inherent limitations that are not easily overcome by technology. In order to broaden the scope of effective TDD therapies it is therefore prudent to search in parallel for additional strategies that would complement the use of ADCs for cancer therapy.

## Alternatives to antibodies as drug carriers in TDD

Several other technologies have been developed to identify TAAs and to use the products as carriers in targeted drug delivery (see Table 3). Arguably the most developed of these is the use of synthetic biology, often based on the bacteriophage display library platform developed by Winter [103]. Newer derivatives of the original platform include the use of different phage strains, bacterial or yeast cells, antibody cDNA, mRNA or random peptide based libraries and various candidate selection protocols. For further details on these systems the reader is referred to recent excellent reviews [2,104-108]. Historically, as phage display systems developed in parallel with technologies for antibody engineering, it is perhaps not surprising that many of the early display platforms focused on combinatorial antibody heavy and light chain variable region gene libraries. The selection of these gene sequence combinations also allowed their direct grafting onto human constant region gene scaffolds, thus expanding the repertoire of full length humanized antibodies [2]. The first FDA approved antibody developed in this way was Adalumumab, an anti-TNFα antibody registered in 2008 for use in the treatment of several autoimmune diseases. Over 20 phage display derived full length TMAs are currently undergoing clinical assessment, mostly for cancer therapy, several of them for hematological cancers. In addition to expanding the binding diversity of full length antibodies, the single-chain "minibodies" generated by these biological systems can be easily linked to drugs as described below. Alongside synthetic biology methods, synthetic chemical techniques also have proven advantageous. A variety of approaches are now available for the generation of combinatorial small molecule, peptide or aptamer libraries for the generation of cancer cell targeting drug carriers [109-113]. Of these strategies, the most developed is that of peptide-drug conjugates and these are discussed further.

## Peptide-Drug Conjugates (PDCs)

During the 1980’s significant research effort and money were invested in the development of peptide therapeutics, but only isolated products reached the marketplace. The first of these was the synthesized peptide hormone luteinizing hormone-releasing hormone (LHRH), (leuprorelin) launched in 1984 by Abbot. Interest waned however, as it became clear that problems with peptide stability and their short blood half lives due to peptidase sensitivity would limit the effectiveness of natural peptides. Continuing progress over the ensuing years in synthetic peptide chemistry and manufacturing processes have largely solved many of the problems to the extent that the field is flourishing today [114]. Recent surveys show that over 50 peptide drugs have been approved for clinical use with several of these drawings over $1billion in annual sales. Another several hundred peptides are in various stages of clinical assessment (http://www.peptidetherapeutics.org/PTF_report_summary_2010.pdf). These data give strong impetus to using peptides as drug carriers.

Several PDCs for cancer therapy have been developed although none have yet received regulatory approval. The most promising of these is GRN1005, an angiopeptin-2-paclitaxol PDC that targets lipoprotein receptor protein-1, a cell surface molecule overexpressed on solid tumor cells. The conjugate is under clinical assessment for treatment of advanced solid tumors, in particular in patients with brain metastases [115]. Examples of other PDCs in development for cancer therapy include candidates for prostate cancer, leukemia, lymphoma and small cell lung cancer using both natural and synthetic peptides conjugated to traditional or novel drugs [116-120]. Peptides as carriers may offer advantages over antibodies and several of these are listed in Table 3.

### Novel target discovery

Peptide display technologies do not rely on the antigenicity of the target molecule. Therefore it is not necessary to develop specialized immunization protocols to generate binding ligands to weak antigens. Also, depending of the technology used, the displayed peptides can be structurally extended from the display platform, allowing them for freedom to bind locations on single molecule or complex target binding sites that might be sterically hindered from more bulky antibodies. This has already generated the discovery of novel peptide ligands of cancer cell surface targets [121].

### Conjugation chemistry

**Figure 2:**
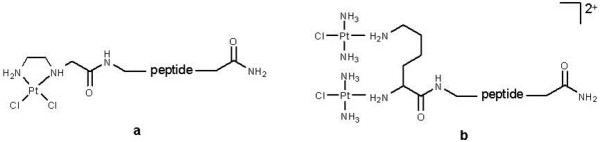
**Mononuclear(a)and Binuclear(b)peptide targeted platinum complexes**, **with targeting peptide** .

The important question of appropriate carrier-linker-drug design and synthesis is easily approached with PDCs. As with full length antibodies, the site of conjugation between peptide and drug molecule can have a profound effect on maintaining peptide binding affinity, drug activity and conjugate stability. However exact knowledge of the peptide sequence and the amino acids responsible for maintaining high binding affinity often allows a higher degree of flexibility in the design of linker length, its composition and conjugation chemistry to the drug. Compared to antibody-linker conjugation, peptide conjugation can then include a wide range of chemistries encompassing amides, carboxylic acid esters, hydrazones, thioethers and carbamates (for more details see [120]. Aside from small molecule drugs, peptides can also be easily conjugated to transition metals. Mononuclear and dinuclear platinum complexes tethered to an α9β1-integrin targeting peptide and a nuclear-localisation peptide have been synthesized using solid-phase synthesis [122] (see Figure 2). The cellular uptake, DNA binding and cytotoxicity of the complexes was monitored in a number of different cell lines. Some of these conjugates exerted remarkable targeted cytotoxicity. However, linkage of platinum complex to antibody is very problematic due to the number of competing Lys amino groups situated across the antibody molecule.

An additional advantage with random peptide libraries is the ability to select combinatorial cyclic peptides which may demonstrate higher selectivity for the target and longer stability than linear peptides because of conformational restriction [123,124]. There is also is flexibility in cyclization strategy [120] in which an amide bond formed between N- and C-termini or the disulphide bond formed between two Cys residues can be engineered outside of the receptor recognition sequence and adjacent to a linker moiety.

### Intracellular uptake

For the most part the molecular targets of anti-cancer drugs are intracellular, a trend likely to continue with the explosion of new information in areas such as signaling pathways, epigenetics, DNA repair mechanisms, cell cycle regulation and mitochondrial metabolism. Therefore it is imperative that carrier-drug complexes induce efficient cellular uptake, preferably through receptor-mediated endocytosis (RME). This will initially traffic the conjugate to endosomes/lysosomes, induce enzymatic or chemical degradation of linker and release active drug into the cytoplasm [125,126]. This process has been extensively studied, with the case of transferrin being only one example [127-130]. It should be noted that peptide carriers can also be taken up non-specifically by pinocytosis [130], however these peptides can be selected out by testing for uptake on appropriate receptor negative control cells. Not only does the RME pathway ensure that the drug payload is delivered intracellularly, but this form of uptake has been shown to one pathway to bypassing the development of drug resistance, a strategy that should be further exploited to enhance the clinical efficacy of drugs already in the clinic [131], (Gellerman et al., submitted).

There are many aspects of carrier-drug uptake/processing pathway to be considered, the first of which is the ability of the carrier itself to bind and activate the receptor in such a way as to induce efficient RME. Peptide display libraries afford a higher statistical chance of presenting at least some candidates in the (novel?) structural orientation to engage the receptor binding pocket correctly and the library platforms more easily lend themselves to high throughput screening for this specification. Furthermore internalizing carrier peptides can be selected from these libraries without prior knowledge of receptor structure or biology. This is important as cell surface receptors vary widely in their cell surface density, turnover time, RME kinetics and recycling and trafficking routes.

### Tumor penetration

Peptide carriers are in the order of a hundredth the mass of antibodies [132] and this is clearly an advantage to overcoming the interstitial tumor pressure which can limit antibody transport into the tumor interior [133,134]. Two examples that emphasize this point are the identification of peptides containing a CendR motif that enhance penetration into tumor tissue and internalization into cells [135] and the use of tumor-vasculature homing peptides containing the CD13 receptor binding motif Asn-Gly-Arg [136]. Finally, while members of the cell-penetrating peptide family can [137], but usually do not demonstrate tumor tissue specificity [138], they can be grafted to a variety of targeting peptides to enhance the efficacy of TDD [139-142].

### Manufacturing

There are now several dozen peptide therapeutics approved for clinical use and the market for therapeutics peptides is estimated to reach $11.5billion by 2013 (BioNest). Over the last decade or so, processes in peptide manufacture have considerably evolved, partly due to the experience of scaling up production from milligram to multi-ton levels [143,144]). These requirements have generated important improvements in both technical and cost efficiency aspects of process design, manufacturing capacity and peptide synthesis [145]. With regards to synthesis, both recombinant and chemical synthetic pathways are available and while traditionally the latter approach had the advantage of being able to insert unnatural amino acids that can increase stability and flexibility in conjugation to drugs, this division is now becoming blurred [146].

### Peptide challenges

As mentioned briefly above, natural peptide drugs are limited by their sensitivity to enzymatic degradation, extensive renal filtration, and nonspecific uptake into tissues such as the liver, all of which resulted in reduced bioavailability and reduced half life in the circulation (minutes) as compared to antibodies (reviewed in [147]. Many of the shortcomings have been overcome by different synthetic chemistry strategies, resulting in the development of peptidomimetics. These include incorporation *D*-enantiomeric amino acids and replacement of alanine residues to increase resistance to gut and serum proteases and coupled the peptide to polyethylene glycol (PEGylation) to reduce liver and kidney elimination [105,147].

## Conclusions: on today's menu – TMAs, ADCs and PDCs

The passive infusion of TMAs directed to cell surface antigens on tumors cells has definitely established itself as a valid strategy for therapy of both hematological and some solid tumors. In several cases, such as with Rituxmab, Transtuzumab and more recently Ipilumumab (Table 1), these treatments have revolutionized the prognosis of patients with certain forms of leukemia, breast cancer and metastatic melanoma respectively. Although not tumor cell specific, the antigens targeted by these antibodies are well characterized and validated and there is accumulated understanding on both the benefits and side effects of targeting them. Concerned for their near-term pipelines, pharmaceutical companies are leveraging this knowledge base to develop a variety of new-generation, genetically modified derivatives, (such as bispecific antibodies and "antibody-like" proteins) aimed at improving both the antibody’s pharmacological profiles and cytotoxic effects, either directly (induction of apoptosis or interruption of growth signaling pathways) or indirectly (through conscription of innate (ADCC, CDC) or T-cell immune effector functions). These developments should expand their clinical potential and are likely to result in the approval of more TMAs in the next few years, particularly for hematological cancers and micrometastatic disease, where limitations of tumor tissue penetration are minimized and immune effector functions can be maximized. While a recent survey found that antibodies to over 90 different TAAs are in clinical study [10], it is doubtful that these candidates will overcome the limitations of TMAs described above with regards to tumor cell specificity, solid tumor penetration and more convenient forms of administration. In addition, the high cost of manufacture remains a point of concern for health care managers.

Like most offspring, full length ADCs inherit some limitations of their naked TMA predecessors listed above and carry new ones of their own but also have the opportunity to overcome others. The noteworthy emphasize of approved and development-stage ADCs targeting hematological cancers (see Table 2) again highlights the limitation of solid tumor penetrability. Interestingly, the need for their uptake in cells contradicts one of the advantages of immunotherapy – the conscription of immune effector functions. On the other hand, the need to induce cellular uptake has prompted the search for new cell surface targets and with improvements in screening and selection techniques it is likely that novel agents will soon emerge.

With the recent advances in peptide chemistry, peptides present a strong alternative to antibodies as drug carriers, even though they still suffer from comparitively reduced half-lives. Whether based on antibody variable region (scFV) or random sequences, the technologies now available offer greater potential for selection of novel peptides with the characteristics required of successful PDCs. Being structurally more flexible than ADCs, peptide building blocks allow the design of non-peptide mimetics with improved characteristics [148], or their conjugation to a dendrimer scaffold comprising several different drugs with different modes of action, These factors become important in light of many clinical studies showing that treatment protocols with drug cocktails are more effective than monotherapies, due to the multiclonal nature of most cancers.

The summary statement of the above discussions is that targeted therapies are poised for a bright future. Naked TMAs, ADCs and PDCs each have advantages and disadvantages and a better understanding of these will allow a more rational selection of mono- or even combination therapies. For example whereas a PDC may be more effective against a tumor mass, an ADC or TMA could be used in combination with the PDC to attack circulating tumor cells or micrometastases. Thus, in the near future physicians, patients and health managers can expect to be provided with a variety of alternatives from which to select a more effective and economic targeted treatment for a particular cancer type.

## Competing interests

Neither of the authors declared any competing interests.

## Authors’ contributions

MF and GG contributed equally to writing the manuscript. Both authors read and approved the final manuscript.
